# Efficacy and Safety of Systemic Thrombolysis and Catheter-Directed Therapy in Pulmonary Embolism: A Narrative Review

**DOI:** 10.7759/cureus.74086

**Published:** 2024-11-20

**Authors:** Henil Upadhyay, Jonathan Barnes, Anna Beattie, John Reicher

**Affiliations:** 1 Respiratory Medicine, Newcastle Upon Tyne Hospitals NHS Foundation Trust, Newcastle Upon Tyne, GBR; 2 Radiology, Newcastle Upon Tyne Hospitals NHS Foundation Trust, Newcastle Upon Tyne, GBR; 3 Interventional Radiology, Newcastle Upon Tyne Hospitals NHS Foundation Trust, Newcastle Upon Tyne, GBR

**Keywords:** percutaneous catheter-directed therapy, pulmonary embolism (pe), reperfusion treatment, systemic thrombolysis, therapeutic anticoagulation

## Abstract

Pulmonary embolism (PE) is the third most frequent cause of acute cardiovascular presentation after myocardial infarction and stroke. The treatment approach for PE consists of hemodynamic and respiratory support, anticoagulation, reperfusion treatment, and vena cava filters. Reperfusion treatment consists of systemic thrombolysis (recombinant tissue-type plasminogen activator, streptokinase, and urokinase); percutaneous catheter-directed therapy (CDT); and surgical embolectomy. CDT is an emerging treatment, with most data derived from randomized controlled trials (RCTs) or case series. Currently, there is a lack of data on clinical efficacy and safety outcomes and a lack of large studies that directly compare CDT with systemic thrombolysis or surgical embolectomy.

This narrative review aims to explore the efficacy and safety of systemic thrombolysis and CDT in pulmonary embolism.

Clinical trials have studied CDT for more than a decade now and have shown that CDT improves the post-procedural right ventricular (RV)/left ventricular (LV) ratio and has a reduced rate of bleeding episodes and all-cause mortality. However, there is a lack of large prospective RCTs studying the effects of CDT in intermediate-high-risk PE patients to determine which patients from this sub-group require CDT both in terms of improving short-term mortality risk and long-term morbidity (such as chronic thromboembolic pulmonary hypertension (CTEPH) and post-PE syndrome). Future clinical trials need to focus on identifying which patient groups will benefit from CDT over anticoagulation and if there are any advantages of using the EkoSonic endovascular system (EKOS) (ultrasound (US)-assisted CDT) over standard CDT. In addition, the scientific community needs to study the healthcare costs of CDT over traditional treatment, which is relevant for public health systems such as the National Health Service (NHS). Lastly, we need standardized guidelines for the use of thrombectomy systems since pulmonary embolism is a complex disease requiring a multifaceted and nuanced treatment approach.

## Introduction and background

Pulmonary embolism (PE) is the third most frequent cause of acute cardiovascular presentation after myocardial infarction and stroke [1]. In terms of epidemiology, the annual incidence of PE ranges from 39-115 per 100,000 population, and an increase in the incidence is seen with increasing age [1]. PE has high mortality rates, and in a European epidemiological model, at least 34% of deaths in venous thromboembolism (VTE) patients were associated with sudden fatal PE before initiation of any therapy, and 59% of deaths were associated with undiagnosed PE [2]. Acute right ventricular strain and failure are critical determinants of clinical severity and prognosis in PE. Therefore, clinical features and signs of right ventricular (RV) failure/hemodynamic instability are indicators of a high risk of mortality [2]. 

Due to the high morbidity and mortality associated with PE, the European Society of Cardiology (ESC) published updated guidelines for the diagnosis and management of acute PE in 2019 [3]. These guidelines classified PE severity into low, intermediate, and high-risk PE based on early (in-hospital or 30-day) mortality risk. The major difference between intermediate and high-risk PE was the absence of overt hemodynamic instability in the background of evident RV strain on imaging and biochemical markers (cardiac troponins). The treatment for both of these groups remains undefined with a high mortality rate, necessitating an aggressive therapeutic approach [4].

Overall, the treatment approach for PE consists of hemodynamic and respiratory support, anticoagulation, reperfusion treatment, and vena cava filters. Reperfusion treatment consists of systemic thrombolysis (recombinant tissue-type plasminogen activator, streptokinase, and urokinase); percutaneous catheter-directed therapy (CDT); and surgical embolectomy [3]. 

For intermediate-risk PE patients, the mortality rate lies anywhere between 3 and 15%, and the current ESC guidelines recommend anticoagulation (without reperfusion techniques) as the first-line therapy in intermediate-risk PE patients. Full-dose systemic thrombolysis is not the first-line treatment in intermediate-risk PE due to the high risk of potentially life-threatening bleeding complications, which outweighs the expected benefits from treatment. Rescue thrombolytic therapy or catheter-directed therapy is reserved for patients who develop hemodynamic instability [3]. 

A 30% mortality rate in high-risk PE patients necessitates the requirement for more effective PE treatment. Primary reperfusion treatment with systemic thrombolysis is the treatment of choice for high-risk PE. If thrombolysis is contraindicated, then percutaneous catheter-directed lysis is an alternative [3]. 

CDT is an emerging treatment, with most data derived from randomized controlled trials (RCTs) or case series. Currently, there is a lack of data on clinical efficacy and safety outcomes and a lack of large studies that directly compare CDT with systemic thrombolysis or surgical embolectomy [3]. 

The aim of this narrative review is to explore the efficacy and safety of systemic thrombolysis and CDT in pulmonary embolism. 

## Review

Methods

We present a narrative review on the efficacy and safety of systemic thrombolysis and catheter-directed therapy in pulmonary embolism. An extensive literature search was conducted on MEDLINE (PubMed) to identify clinical trials conducted in pulmonary embolism to date. The following Medical Subject Headings (MeSH) terms and their synonyms were used: catheter-directed therapy; clinical trials; efficacy; safety profile; systemic thrombolysis; pulmonary embolism; adverse effects and adverse events of special interest. 

Clinical trials were categorized according to systemic thrombolysis or catheter-directed therapy, and initial screening was done by authors HU and JB to assess the suitability of the articles for inclusion in the review. All phase-2 and phase-3 clinical trials involving human subjects were included in the review, while animal studies were excluded. Each selected study was read in-depth to understand the study setup, the patient population, primary and secondary endpoints, and the key findings from the trial by authors HU and JB. Data was extracted from these trials and presented in a concise format. Authors AB and JR contributed to reviewing the initial draft, suggesting further references, making necessary revisions, and approving the final draft of the manuscript.

**Figure 1:**
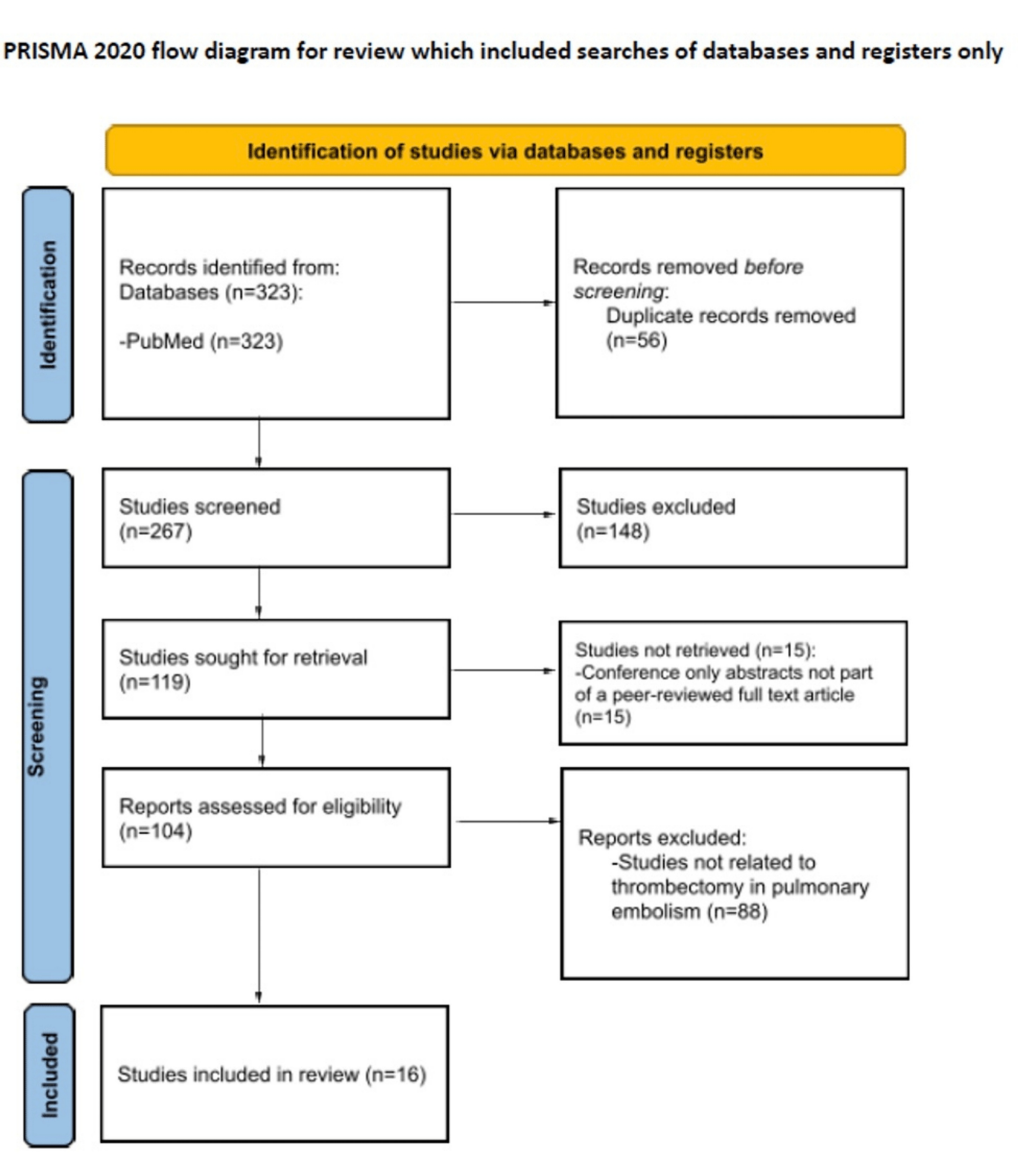
PRISMA flow diagram This PRISMA details our search and selection process applied during the review [5]. PRISMA: Preferred Reporting Items for Systematic Reviews and Meta-Analyses This work is licensed under CC BY 4.0.

The Preferred Reporting Items for Systematic Reviews and Meta-Analyses (PRISMA) guidelines were used while performing the literature search and are shown in Figure 1 [5].

Review

Thrombolytic therapy

PEITHO-1 Clinical Trial 

The pulmonary embolism thrombolysis (PEITHO-1) trial was designed to study the efficacy and safety of systemic thrombolysis (with single-bolus dose tenecteplase) in addition to standardized anticoagulation (with unfractionated heparin (UFH)) in patients with intermediate-risk PE. This trial was designed since previous trials studying fibrinolysis versus heparin had recruited less than 1000 patients, and hence a trial with a large study population was required [6]. 

This was a multicenter, double-blind, placebo-controlled RCT wherein intermediate PE patients (n=1006) were randomized to receive either a weight-based intravenous bolus of tenecteplase or a matching placebo (n=506 and 500, respectively) given over five to 10 seconds. UFH was given as an intravenous bolus to both groups immediately after randomizing patients [6].

The primary efficacy outcome in this trial was defined as a clinical composite of death within seven days of randomization. Similarly, safety outcomes were defined as intracranial or extracranial bleeding within seven days and serious adverse events (SAE) within 30 days of treatment [6]. 

Thirteen patients (2.6%) in the tenecteplase arm achieved primary efficacy outcomes (death within seven days) compared to 28 patients (5.6%) in the placebo group. Similarly, eight patients (1.6%) in the tenecteplase group required mechanical ventilation compared to 15 patients (3%) in the placebo group. In terms of 30-day follow-up mortality, death occurred in 12 patients (2.4%) in the tenecteplase group versus 16 (3.2%) in the placebo group [6]. 

Major bleeding was seen in 58 patients (11.5%) of the tenecteplase group compared to 12 patients (2.4%) in the placebo group. In this trial, older patients had a higher rate of major extracranial bleeding than younger patients. However, this was not significant statistically [6].

Results from the PEITHO-1 trial show that single-dose, weight-based systemic tenecteplase significantly reduces the incidence of death or hemodynamic decompensation in the treatment group as compared to the placebo (2.6% vs. 5.6%). This trial showed that prompt fibrinolysis therapy reduces the risk of hemodynamic decompensation/death in patients with intermediate-risk PE [6].

However, fibrinolytic therapy was associated with an increased rate of hemorrhagic stroke (2% vs. 0.2%, treatment vs. placebo group, respectively) and major extracranial hemorrhage (6.3% vs. 1.2%, treatment vs. placebo group, respectively). This trial also showed that younger patients were at a lower risk of bleeding, but this was not statistically significant. This is similar to a review by Mikkola et al., which demonstrated that patients are at a higher risk of bleeding complications with an increasing age and number of co-morbid conditions [6,7]. 

The PEITHO-1 trial concluded that single-dose tenecteplase reduces hemodynamic decompensation and death in intermediate-risk PE patients; however, this comes with an increased risk of intracranial and other major bleeding. Hence, caution is warranted when considering tenecteplase in this subset of PE patients [6].

The PEITHO-1 trial established the efficacy and long-term clinical outcomes of systemic thrombolytics. However, this was achieved with an increased risk of bleeding complications. In addition, the PEITHO-1 trial was not designed to identify whether early systemic thrombolysis prevents chronic thromboembolic pulmonary hypertension (CTEPH) in intermediate-high-risk PE patients [6].

Since the PEITHO-1 trial did not focus on long-term follow-up, a protocol amendment was put in after which patients randomized in the PEITHO-1 trial were followed up 24 months after randomization. Analysis from this follow-up data shows that thrombolysis had no impact on the long-term mortality rate (20.3% vs. 18% in the tenecteplase and placebo groups, respectively). Similarly, 85% and 96% of patients in the tenecteplase and placebo arms, respectively, had chronic pulmonary hypertension. The diagnosis of CTEPH was confirmed in 2.1% and 3.2% of the patients randomized to tenecteplase and placebo, respectively [6].

Limitations of these long-term follow-up results include participation at fewer sites as compared to the original trial, the inability to identify the true incidence of CTEPH after PE, and whether thrombolysis would help in preventing this complication. In addition, clinical and echocardiographic examinations were not performed on all survivors [8].

PEITHO-2 Clinical Trial 

The PEITHO-2 trial was designed after the post hoc analysis of the PEITHO-1 trial showed persistent RV dysfunction and incomplete echocardiographic recovery on a long-term follow-up of 37 months after acute PE [9]. This trial aimed to study whether an early switch from parenteral heparin to oral anticoagulant dabigatran was safe and effective in intermediate-risk PE patients [10,11]. 

This was a single-arm study wherein PE patients were treated with either low molecular weight heparin (LMWH) or UFH for 72 hours after diagnosis of PE before switching to oral dabigatran 150 mg twice daily. The primary outcome of this study was recurrent symptomatic VTE or PE-related death within six months of treatment [10].

Around 402 patients were enrolled in this study, out of which seven (2%) patients developed recurrent symptomatic VTE or PE-related death, 11 (3%) had major bleeding, and eight (2%) died from any cause. In addition, RV dysfunction was present in 338 patients (84%) at baseline visit [10,11].

This study showed that two-thirds of patients who were switched to dabigatran in the early days of their recovery (day three) had normalized RV function on echocardiography within six days, and this remained normalized throughout the first six months. However, at least one in four patients had an abnormal echocardiography finding, suggesting the persistence of RV dysfunction and the need for long-term follow-up [11].

PEITHO-3 Clinical Trial 

The PEITHO-3 trial is being conducted to assess the efficacy and safety (death, hemodynamic decompensation, recurrent PE) of reduced-dose systemic alteplase vs. standard care heparin in patients with acute intermediate-high-risk PE after 30 days of randomization. This is a randomized, double-blind, placebo-controlled RCT with long-term follow-up. Patients in the experimental arm received tenecteplase 0.6 mg/kg (with a maximum dose not exceeding 50 mg) as a 15-minute intravenous infusion [12].

Similar to the PEITHO-1 trial, the primary outcome in this trial is the clinical composite of death from any cause, hemodynamic decompensation, or recurrent PE within 30 days of randomization. The focus of this trial is to identify safer low-dose reperfusion modalities since systemic thrombolysis is an affordable and feasible treatment modality in many parts of the world [12]. 

The MOPETT Trial 

The moderate pulmonary embolism treated with thrombolysis (MOPETT) trial was conducted to evaluate the role of a lower ‘safe dose’ of tissue plasminogen activator (tPA) with the hypothesis that lungs are highly sensitive to thrombolysis as they are the only organ to receive entire cardiac output [13]. 

In this trial, patients with moderate PE were defined as those with clinical features of PE with CT pulmonary angiogram (CTPA) involvement of >70% involvement of thrombus in ≥2 lobar/left/right pulmonary arteries or a ventilation/perfusion (V/Q) scan showing V/Q mismatch in ≥2 lobes. The ‘safe dose’ of tPA was defined as ≤50% of the standard dose (100 mg) or 0.5 mg/kg (if weight <50 kg), which was given as a 10-mg bolus intravenously within one minute, followed by the remaining 40 mg within two hours. Patients in the control group received anticoagulation (UFH or enoxaparin) alone [13].

Around 121 patients were randomized to either the thrombolysis (n=61) or control (n=60) group. At the end of the follow-up period (28±5 months), nine patients each (16%) in the thrombolysis group had pulmonary hypertension and pulmonary hypertension plus recurrent PE. In contrast, 32 patients (57%) in the control group had pulmonary hypertension, and 35 (63%) had pulmonary hypertension plus recurrent PE on follow-up. No patients in the thrombolysis group had recurrent PE compared to three (5%) patients in the control group [13].

In terms of hospital stay, patients in the control group (4.9±0.8) had a longer stay as compared to the thrombolysis group (2.2±0.5 days). This is similar to the results seen in the PEITHO trials. Similarly, total mortality was lower in the treatment group (1.6%) as compared to the control group (5%). This study shows that a 50% dose reduction of tPA was safe and effective for treating moderate PE [9,10,13].

Catheter-directed therapy (CDT)

Catheter-directed therapy is based on mechanical and/or pharmaco-mechanical thrombus fragmentation and aspiration of the thrombus via the insertion of a catheter through femoral access. Multiple clinical trials have shown CDT to be an effective therapy in both intermediate- and high-risk PE patients with a low risk of bleeding complications, such as hemorrhagic stroke and life-threatening bleeding.

Ultrasound-Assisted Catheter-Directed Thrombolysis (USAT)

Ultrasound-assisted catheter-directed thrombolysis (USAT) combines conventional catheter-directed thrombolysis with high-frequency, low-power ultrasound. Ultrasound is postulated to increase the permeability of thrombolytic drugs but causes reversible disaggregation and separation of fibrin fibers [14]. 

There are three clinical trials, namely the ULTIMA trial, SEATTLE-II trial, and OPTALYSE PE, of USAT. However, these trials have different comparison groups [14-16]. The SUNSET PE is another trial that compares the efficacy and safety of CDT vs. USAT [17].

ULTIMA trial: In the ultrasound accelerated thrombolysis of pulmonary embolism (ULTIMA) trial, 59 patients with intermediate-risk PE were randomized to either UFH (80 IU/KG) and the USAT regimen of 10 mg recombinant tPA over 15 hours per treated lung via the EkoSonic endovascular system or UFH alone (n=30 vs. 29, respectively). The primary endpoint for this trial was the difference in the right/left ventricular (R/LV) ratio from baseline to 24 hours post-treatment. A follow-up visit was conducted at 90 days with repeated echocardiography [14]. 

The mean RV/LV ratio was reduced from 1.28±0.19 at baseline to 0.99±0.17 at 24 hours in the USAT group, whereas the mean RV/LV ratio was reduced from 1.20±0.14 at baseline to 1.17±0.20 at 24 hours in the heparin control group. On 90-day follow-up, echocardiography showed far better improvement in the RV function in the USAT group as compared to the heparin group. The RV/LV ratio in the USAT and heparin groups at 90 days was 0.92±0.15 and 0.96±0.16, respectively [14]. 

No major bleeding complication (defined as >2 g/dL drop in hemoglobin levels or bleeding involving a critical site) was observed after the standardized USAT regimen. There were only three minor bleeding episodes in the USAT group and one in the heparin group. There was no episode of hemodynamic decompensation or recurrent VTE among the 59 patients [14].

The ULTIMA trial showed that a standardized USAT regimen was superior to UFH anticoagulation alone in reversing right ventricular dilatation without an increase in major bleeding complications at 24 hours post-procedure. In addition, there was an early improvement in all assessed echocardiographic parameters in the USAT group, while the same was not seen in the heparin group [14]. 

However, this study did not study the effect of ultrasound, as there was a lack of a thrombolysis control group without ultrasound. In addition, the post-procedure residual embolic burden was not studied. Further trials are required to answer these questions [14]. 

The SEATTLE II clinical trial: The concept of the SEATTLE II study is in line with the ULTIMA trial, wherein pharmaco-mechanical CDT combines fibrinolytic therapy with mechanical disruption of thrombus using ultrasound waves. This study was aimed at studying whether low-dose fibrinolysis was effective in reversing RV dysfunction in acute massive or submassive PE treated with ultrasound-facilitated, catheter-directed, low-dose fibrinolysis [15].

This was a single-arm study wherein 150 patients were randomized in this study to either 24 mg tPA (1 mg/hr for 24 hours with a unilateral catheter or 1 mg/hr for 12 hours with bilateral catheters). The EkoSonic endovascular system was used to deliver ultrasound therapy [15]. 

The primary efficacy outcome was defined as a change in the chest CT-measured RV/LV ratio within 48 hours of procedure initiation. In addition, the primary safety outcome was defined as major bleeding within 72 hours of procedure initiation [15].

In this trial, a 25% decrease in CT-measured RV/LV diameter ratio, from 1.55 at baseline to 1.13 at 48 hours after initiation of the procedure, was seen. In addition, a 30% decrease in pulmonary arterial systolic pressure by the end of the procedure (from 51.4 mmHg to 37.5 mmHg) and a 30% decrease in pulmonary artery angiographic obstruction over 48 hours (from 22.5 to 15.8) were observed [15]. 

In terms of safety outcomes, none of the patients suffered from intracranial hemorrhage after the procedure, while 15 patients suffered from other major bleeding within 30 days. Three patients (2%) died while in hospital, and four patients (2.7%) died within 30 days of the procedure [15]. 

An exploratory analysis was conducted after the completion of the SEATTLE II trial to assess the risk factors for major bleeding. This was defined as Global Utilization of Streptokinase and Tissue Plasminogen Activator for Occluded Coronary Arteries (GUSTO) moderate or severe/life-threatening bleeding events [17,18]. 

Results from this analysis show that major bleeding was reported in 10% of patients undergoing catheter-directed fibrinolysis. Nearly a quarter of these episodes were associated with vascular access, and the factors most strongly associated with increased risk of bleeding included diagnosis of massive PE and multiple attempts at obtaining venous access. This is similar to the results obtained from the PEITHO trial for systemic fibrinolytic, wherein major bleeding episodes were seen in 11.5% of patients. GUSTO moderate and severe bleeding episodes were seen in 9.3% and 0.7% of patients, respectively [6,17-19]. 

Even though the concept of both the ULTIMA and SEATTLE II studies was the same, a lower dose of tPA (20 mg) was used in the ULTIMA study, and only 30 patients in the ULTIMA trial had sub-massive PE, whereas the SEATTLE II study had 150 patients with sub-massive or massive PE. Since the SEATLE II study included patients with massive PE, this led to higher risk and therefore a higher percentage of patients experiencing bleeding episodes as compared to the ULTIMA trial (10% vs. 0) [14,15]. 

A major limitation of the SEATTLE II study was that there was no control arm to compare the efficacy of low-dose fibrinolysis with systemic thrombolysis or anticoagulation alone. Similar to the ULTIMA trial, this study did not study the effects of ultrasound as there was no comparator arm [15]. 

OPTALYSE PE trial: A randomized trial of the optimum duration of acoustic pulse thrombolysis procedure in acute intermediate-risk pulmonary embolism (OPTALYSE PE) trial is based on the utilization of ultrasound-facilitated catheter-directed thrombolysis (USCDT) in symptomatic intermediate-risk PE patients using the EkoSonic endovascular system. This study hypothesized that lower doses of tPA and shorter duration of USCDT than in the previous trials would result in RV function improvement in intermediate-risk PE patients [14-16]. 

101 patients were enrolled and randomized to either of the four arms (Table 1).

**Table 1:** Treatment regimen in OPTALYSE PE trial tPA: tissue plasminogen activator; USCDT: ultrasound-facilitated catheter-directed thrombolysis; OPTALYSE PE: optimum duration of acoustic pulse thrombolysis procedure in acute intermediate-risk pulmonary embolism

Arm	USCDT	tPA dose per catheter	tPA range
Arm 1 (n=28)	USCDT *2 hours	2 mg/hr	4-8 mg
Arm 2 (n=27)	USCDT *4 hours	1 mg/hr	4-8 mg
Arm 3 (n=28)	USCDT *6 hours	1 mg/hr	6-12 mg
Arm 4 (n=18)	USCDT *6 hours	2 mg/hr	12-24 mg

All patients received therapeutic anticoagulation with heparin, irrespective of the arm. The heparin dose was reduced to 300-500 U/h during tPA infusion and increased to full therapeutic dosing after USCDT. 

The primary and secondary efficacy endpoints in this trial were defined as the change in RV/LV ratio from baseline to 48±6 hr after initiation of therapy and the change in embolic burden from baseline as measured by the modified Muller score, respectively. A low dose of thrombolytic therapy (4-12 mg per lung) and shorter infusion times (two to six hours) were associated with statistically significant improvement in RV/LV ratio (by 25%) as compared to baseline in all four arms. The modified Muller score also improved statistically with an increase in tPA dose and infusion duration in all arms [16]. 

The primary safety outcome was defined as the frequency of major bleeding events within 72 hours of treatment, symptomatic recurrent PE, and patient mortality. The major bleeding rate was 4% and 3.6% in the intention to treat (ITT) and modified-per-protocol (mPP) groups, respectively. When compared across different arms, arm 4 (tPA range 12-24 mg) was associated with a higher rate of major bleeding events [16].

The overall ICH event rate across all three pharmaco-mechanical trials (ULTIMA, SEATTLE II, and OPTALYSE PE) was 0.72%, and only one ICH event (0.36%) was attributable to USCDT [14-16]. 

This study showed that a low dose of tPA and shorter infusion time result in statistically significant improvement in RV/LV diameter ratio with a low rate of major bleeding events. One of the limitations of this study was the small number of patients in each arm and the absence of a heparin control group. However, this lack of a heparin control group was previously addressed in the ULTIMA trial [14,16].

SUNSET sPE trial: The standard vs. ultrasound-assisted catheter thrombolysis for submassive pulmonary embolism (SUNSET sPE) is the only randomized, single-blind clinical trial that compares the efficacy and safety of USAT vs. CDT in patients with submassive (intermediate-risk) PE since it is unproved whether USAT using the EkoSonic endovascular system is superior to standard CDT [17]. 

The primary efficacy outcome in this study was defined as the change in the CT obstruction score using the refined modified Muller scoring system. Secondary efficacy outcomes included change in the RV/LV ratio from baseline at 48 hours post-procedure, ICU and hospital stay, death, hemodynamic decompensation, bleeding episodes, and SAE up to 90 days after randomization [17]. 

Around 82 patients were randomized into either the USAT (n=40) or standard catheter-directed thrombolysis (SCDT) (n=41) group. In terms of primary efficacy endpoint, pre- and post-intervention CT scans were performed, which showed a mean raw PA thrombus score reduction from 31±4 at baseline to 22±7 postintervention in the USAT group. Similarly, in the SCDT group, the mean raw PA score reduced from 33±4 at baseline to 23±7 postintervention. However, there was no statistical difference in the mean raw PA thrombus score reduction between the two groups. The CT obstruction index reduced from 71±8% to 50±17% (baseline to postintervention) in the USAT group and from 73±7% to 51±15% in the SCDT group (baseline to postintervention). This shows that patients undergoing USAT did not have improved pulmonary arterial thrombus reduction as compared to SCDT [17]. 

Secondary endpoint analysis showed the mean RV/LV ratio reduction from 1.5±0.3 at baseline to 1.2±0.2 postintervention in the USAT group. The SCDT group showed a reduction in the mean RV/LV ratio from 1.7±0.4 at baseline to 1.1±0.2 (postintervention) in the SCDT group. The SCDT group showed superior RV/LV ratio reduction as compared to the USAT group. The average ICU stay was shorter in the SCDT group as compared to the USAT group (4.6± 1.8 days vs 7.7±8.7 days) [17]. 

There were no major or minor bleeding events in the SCDT group, whereas the USAT group saw two major and three minor bleeding events. This trial showed one of the lowest major bleeding rates (2.5%) in the SCDT group [17]. 

In summary, SCDT was associated with a 30% reduction in RV/LV ratio within 48 hours of therapy. There was no added clinical benefit of USAT in RV/LV ratio reduction as compared to SCDT. However, there is limited evidence of the superior efficacy of USAT vs. SCDT due to the lack of prospective comparative outcomes data and with current evidence derived mostly from small, equivocal retrospective studies [17]. 

HI-PEITHO trial: The higher-risk pulmonary embolism thrombolysis (HI-PEITHO) is an ongoing multicenter RCT designed with a primary objective to assess whether USCDT plus anticoagulation is associated with a significant reduction in the composite outcome of PE-related mortality, cardiorespiratory decompensation or collapse, or recurrence of PE within seven days of randomization compared to anticoagulation alone. In the USCDT arm, patients will be administered alteplase using the EkoSonic endovascular system, whereas patients in the control arm will be administered subcutaneous LMWH or intravenous UFH at a therapeutic dose [20]. 

This is a unique trial comparing the efficacy and safety of USCDT with heparin anticoagulation and is expected to be completed by December 2023. Results from this trial are expected to provide further evidence of the superiority of either USCDT or heparin therapy in PE patients [20].

Table 2 summarises the ultrasound-assisted CDT trials in pulmonary embolism.

**Table 2:** Ultrasound-assisted CDT trials in pulmonary embolism PA: pulmonary artery; RV/LV: right ventricular/left ventricular; tPA: tissue plasminogen activator; CDT: catheter-directed therapy; UFH: unfractionated heparin; USAT: ultrasound-assisted catheter-directed thrombolysis; USCDT: ultrasound-facilitated catheter-directed thrombolysis

Clinical trial	Structure of study	PE classification	Efficacy outcome in active group	Safety outcome in active group
ULTIMA [13]	Double arm (USAT vs. UFH)	Intermediate risk	Reduction in RV/LV ratio from 1.28±0.19 at baseline to 0.99±0.17 at 24 hours	No major bleeding complications in the USAT group
SEATTLE II [14]	Single arm (low dose tPA)	Massive and sub-massive	Reduction in mean RV/LV diameter ratio from 1.55 to 1.33	10% rate of major bleeding episodes within 30 days
OPTALYSE PE [15]	4 arms (USCDT-tPA)	Intermediate risk	25% improvement in RV/LV diameter ratio in all arms	Overall, 5% rate of major bleeding within 72 hours
SUNSET sPE [16]	Double arm (USAT vs CDT)	Intermediate risk	No statistically significant difference in mean PA raw thrombus score reduction between the 2 arms	Major and minor bleeding episodes in USAT arm; no bleeding episode in SCDT arm
HI-PEITHO [19]	Double arm (USCDT vs UFH)	Intermediate-high risk	Currently active trial	Currently active trial

Percutaneous Mechanical Thrombectomy 

The FLARE trial: The FlowTriever pulmonary embolectomy clinical study (FLARE) trial included intermediate-risk PE patients who were hemodynamically stable but showed evidence of right ventricular strain on imaging and biochemical markers. This was a prospective, single-arm, multicenter investigational device exemption trial wherein patients with intermediate-risk PE were treated with the FlowTriever retrieval/aspiration system [21]. 

The FLARE trial was unique as it reports the use of CDT in intermediate-risk PE patients, which was not studied in trials before. This was because the current PE guidelines recommend CDT in hypotensive patients only who have high bleeding risk, have failed thrombolysis, or have shock [21]. 

The primary efficacy endpoint in this trial was defined as a change in RV/LV ratio from baseline to 48 hours post-procedure assessed using CTPA, and the primary safety endpoint was defined as the major adverse event (MAE) rate within 48 hours of treatment. These MAEs included major (life-threatening or disabling) bleeding episodes, treatment-related clinical deterioration or pulmonary vascular injury or cardiac injury, and device-related death [18]. A secondary safety endpoint was defined as all primary safety events with the addition of morality, device-related SAE, and symptomatic recurrence of embolism within 30 days of the procedure [21].

In this study, 106 patients were enrolled, with the majority of patients presenting with bilateral PE (90.4%) and 73 patients (70.2%) having residual deep vein thrombosis (DVT) [21]. 

The average pre-procedural and post-procedural RV/LV ratio was 1.56±0.34 mm (n=104) and 1.15 ±0.25 mm (n=101). An average 25.1% post-procedural reduction in RV/LV ratio was seen in 101 patients. A significant difference in post-procedural mean PA pressure (mPAP) was seen as compared to pre-procedure (29.8 mmHg vs. 27.8 mmHg), primarily in those patients who had pulmonary hypertension at baseline. However, this difference in mPAP was not seen in patients with a normal baseline mPAP [21].

In terms of safety outcomes, only four (3.8%) patients experienced MAEs within 48 hours of the procedure. This rate was significantly lower than the performance goal of <25%. These MAEs included major bleeding events, pulmonary vascular injury, respiratory deterioration, and ventricular fibrillation. These MAEs were adjudicated as procedure-related but not device-related. Fourteen patients (13.2%) experienced SAEs within 30 days of the procedure. No patients suffered from post-procedure ICH, and the major bleeding rate in this trial was reported as 0.9%. The average length of intensive care unit (ICU) stay in this trial was 1.5 days, and 43 patients (41.3%) did not require any ICU stay at all [21]. 

Results from this trial show that percutaneous mechanical thrombectomy has acceptable clinical effectiveness and safety profile in intermediate-risk PE patients. The FlowTriever system met both its efficiency (25.1% RV/LV ratio reduction) and safety performance goal (composite MAE rate of 3.8%) [21]. 

However, the limitations of this study were the lack of a comparator arm and the non-randomized design. In addition, there was no long-term follow-up for these patients; hence, there is no data available to compare the long-term effects of CDT [21].

The FLAME study: The FLAME is a unique multicenter, prospective, observational study of high-risk PE patients wherein the treatment selection was at the discretion of the physician and no randomization was performed. It is the largest prospective study in high-risk PE patients wherein patients were enrolled in either of the two arms: the FlowTriever arm (treated with FlowTriever system) (n=53) or the context arm (treated with other non-FlowTriever therapies) (n=61). The FlowTriever system is a large-bore mechanical thrombectomy system. In the context arm, the majority of the patients were treated with systemic thrombolytic therapy (n=42, 68.9%), followed by anticoagulation alone (n=14, 23%), catheter-directed non-FlowTriever thrombolytic therapy (n=4,6.6%) and surgical embolectomy (n=1, 1.6%) [22]. 

The primary safety endpoint in this study was defined as a composite of all-cause in-hospital mortality, bailout to an alternate thrombus removal strategy, clinical deterioration, and major bleeding. The composite primary endpoint was achieved in 17% of patients in the FlowTriever arm (performance goal 32%, p<0.01) as compared to 63.9% in the contrast arm. The hospital mortality rate was significantly lower in the FlowTriever arm as compared to the contrast arm (1.9% vs. 29.5%, respectively) [22]. 

This low mortality rate in the FlowTriever arm can be attributed to the immediate effects of thrombus removal on pulmonary perfusion, right ventricular strain, and hemodynamic instability. These results have been reproduced in other trials, such as the FLASH registry, wherein the all-cause mortality in high-risk PE group patients was 0.3% and 0.8% at 48-hour and 30-day follow-ups, respectively [22,23]. 

With regards to other outcomes, a bailout to alternate thrombus strategy occurred in 3.8% of FlowTriever patients and 26.2% of context arm patients; clinical deterioration was seen in 15.1% of FlowTriever arm patients and 21.3% of context arm patients. Major bleeding occurred in 11.3% of FlowTriever arm patients and 24.6% of the context arm patients. In addition, no ICH was seen in the FlowTriever arm; however, the same was seen in the context arm (4.8%) [22]. 

Results from this trial show that patients in the context arm who received non-FlowTriever therapies required qualitatively intense cardiac arrest, vasopressor therapy, and stage E cardiogenic shock. However, since this was an observational study and therapy was at the discretion of the treating physician, there could have been a selection bias [22]. 

Since this was an observational study, patient presentation and treatment pathways of this trial closely resemble those of real-life scenarios and are influenced by various factors such as PE response team consultations, PE location, availability of specialists, and catheterization laboratory resources. This means that the low mortality rate observed in this trial can be replicated in other centers, especially in high-risk PE patients, and can help improve survival rates if provided as standardized care [22].

EXTRACT-PE trial: A prospective, multicenter trial to evaluate the safety and efficacy of the Indigo aspiration system in acute pulmonary embolism (EXTRACT-PE trial) was the first prospective single-arm trial studying the efficacy and safety of the Indigo aspiration system in submassive PE without the use of thrombolytic drugs [24].

The primary efficacy endpoint in this study was the difference between the baseline and the 48-hour RV/LV diameter ratio, and the primary safety endpoint was defined as the rate of MAE at 48 hours, a composite of device-related death, major bleeding, and device-related SAE. Similarly, the secondary endpoints at 48 hours were defined as the rate of device-related death, major bleeding, clinical deterioration, pulmonary vasculature, or cardiac injury [24]. 

Similar to the FLAME study, which was focused on high-risk PE patients and resembled real-life scenarios, this study was focused on submassive PE patients, with 118 out of 119 patients being diagnosed with submassive PE. The majority (n=111, 93.3%) of the patients in this trial had bilateral PE, while the remaining 6.7% (8/119 patients) had unilateral PE, which is similar to the FLARE trial [24]. 

The EXTRACT-PE trial showed a significant reduction (27.3%) in the RV/LV ratio at 48 hours post-procedure (from 1.47 to 1.04) and an average 7.9% post-procedure reduction in the systolic pulmonary artery pressure. This significant mean reduction in RV/LV ratio (0.43) at 48 hours post-procedure is similar to that seen in the SEATTLE II study (0.42) and more than that seen in the FLARE trial (0.38). Around 89.6% (n=103) of patients in this trial had a significant RV/LV ratio reduction without suffering from any adverse event or clinical deterioration. One notable difference was the device-specific treatment time, which was faster in the EXTRACT-PE trial (median 37 min) than in the FLARE trial (mean 57 min) [7,15,21,24]. 

The major adverse event rate was only 1.7% after the use of the Indigo Aspiration System, with no patients suffering from hemorrhagic strokes or requiring blood transfusions. The major bleeding rate (1.7%) in this trial was lower than in thrombosis trials (1% in the FLARE trial, 11.5% in the PEITHO trials, and 10% in the SEATTLE II trials). Limitations of this study included lack of long-term follow-up, randomization, and comparator arm [7,15,21,24]. 

CANARY clinical trial: The catheter-directed thrombolysis vs. anticoagulation monotherapy in patients with acute intermediate-high-risk pulmonary embolism (CANARY) trial was designed to study the long-term benefits of CDT in patients with intermediate-to-high-risk PE at three-month follow-up. This was an open-label, masked-end point randomized clinical trial with patients administered either conventional CDT (cCDT) and anticoagulation or anticoagulation alone [25]. 

The primary study outcome was defined as the percentage of patients with RV/LV ratio >0.9 at three-month follow-up. Similarly, the secondary outcomes were defined as the percentage of patients with RV/LV ratio >0.9 at 72 hours after randomization, those with unrecovered RV at three-month follow-up, and the three-month rate of all-cause mortality [25]. 

Around 94 patients were randomized to either cCDT plus anticoagulation (n=48) or anticoagulation monotherapy (n=46). At three-month follow-up, there was no significant difference in the proportion of patients with RV/LV ratio >0.9 between the cCDT and the monotherapy group (4.3% vs 12.8%, respectively). However, the median RV/LV ratio was significantly lower in the cCDT group as compared to the monotherapy group at follow-up (0.7 vs. 0.8%, respectively) [25].

In terms of secondary outcomes, fewer patients in the cCDT group had an RV/LV ratio >0.9 at 72 hours post-randomization as compared to the anticoagulation monotherapy group (27% vs. 52.1%, respectively). Similarly, the median RV/LV ratio was lower in the cCDT group at 72 hours post-randomization as compared to the anticoagulation group (0.8 vs. 0.9, respectively). The rate of unrecovered RV function was lower at three-month follow-up with cCDT compared with anticoagulation monotherapy (6.2% vs. 28.2%, respectively). The composite three-month all-cause mortality was lower in the cCDT group as compared to the monotherapy group (4.2% vs. 17.3%, respectively). Only one patient in the cCDT group had a major bleeding event, and there were no fatal bleeding events or ICH [25].

Since this trial was prematurely terminated due to the COVID-19 pandemic, the trial was underpowered to detect a statistically significant difference in the efficacy and safety of cCDT with anticoagulation monotherapy [25].

PEERLESS I trial: The PEERLESS clinical trials are the largest RCTs of mechanical trials to date. The PEERLESS I trial is a prospective, multicenter RCT aimed at comparing the clinical efficacy of large-bore mechanical thrombectomy vs. CDT in patients with hemodynamically stable PE. This study is currently enrolling patients who will be randomized to either large-bore mechanical thrombectomy or CDT (1:1), and it is the first RCT to compare two different interventional treatment stratifies for hemodynamically stable PE patients [26]. 

Patients in the mechanical thrombectomy arm will be treated with the Inari FlowTriever system, while patients randomized to the CDT arm will be treated with any of the commercially available CDT systems at the discretion of the treating physician, including the conventional CDT and ultrasound-assisted CDT [26].

In this study, patients will have a follow-up 24 hours post-procedure at the time of discharge from the hospital and 30 days post-discharge. The primary endpoint in this study is a composite endpoint constructed as a win ratio, which is a hierarchy of the following outcomes assessed either seven days post-procedure or at hospital discharge, whichever is earlier: all-cause mortality, ICH, major bleeding event, clinical deterioration and/or bailout to alternate thrombus removal strategy and ICU stay. Secondary endpoints include clinically relevant non-major and minor bleeding events defined as per the study protocol [26].

The results of this study are expected to be published by late 2024 or early 2025 and will help in understanding the effects of large-bore mechanical thrombectomy devices and CDT in PE patients. However, this study does not include an anticoagulation treatment arm and hence will not be able to compare whether CDT is superior to anticoagulation treatment or which patients are more likely to benefit from CDT [26].

PEERLESS-II trial: The PEERLESS-II is the first multicenter RCT that aims to evaluate the outcome in intermediate-risk PE patients randomized to treatment with large-bore mechanical thrombectomy + anticoagulation vs. anticoagulation alone. This trial is currently enrolling up to 1200 patients across 100 sites globally. This trial aims to evaluate patient-centric clinical and functional endpoints as opposed to surrogate measures related to RV function used in other trials. Data will be collected 48 hours post-procedure at one-month and three-month follow-up visits [27]. 

The primary endpoint is a hierarchical win ratio assessing short-term mortality, clinical deterioration, hospital readmission, bailout therapy, and dyspnea outcomes across both arms. PEERLESS II complements and extends the efforts of the previous trials comparing CDT and anticoagulation in intermediate-risk PE patients (HI-PEITHO and PE-TRACT study). In addition, this is the only study that exclusively focuses on mechanical thrombectomy as an initial treatment for intermediate-risk PE [20,27]. 

Table 3 summarises the percutaneous mechanical thrombectomy trials in pulmonary embolism.

**Table 3:** Percutaneous mechanical thrombectomy trials in pulmonary embolism CDT: catheter-directed therapy; PE: pulmonary embolism; RV/LV: right ventricular/left ventricular; OPTALYSE PE: optimum duration of acoustic pulse thrombolysis procedure in acute intermediate-risk pulmonary embolism

Clinical trial	Structure of study	PE classification	Efficacy outcome in active group	Safety outcome in the active group
FLARE trial [21]	Single arm (FlowTriever system)	Intermediate risk	25.1% post-procedural reduction in RV/LV radio	Major adverse events in 3.8% of patients
FLAME trial [22]	Double arm (FlowTriever vs. non-FlowTriever system)	High risk	No efficacy outcome measures	1.9% vs 29.5% all-cause mortality rate (FlowTriever vs. non-FlowTriever system)
EXTRACT PE trial [24]	Single arm (Indigo Aspiration system)	Sub-massive	27.3% mean reduction in RV/LV ratio 48 hours post-procedure	1.7% major adverse events within 48 hours
CANARY trial [25]	Double arm (CDT vs. anticoagulation)	Intermediate-high risk	No significant difference in primary efficacy outcome between the two groups at 3 months follow-up	4.2% all-cause mortality rate in the CDT arm vs 17.3% in the anticoagulation arm
PEERLESS I trial [26]	Double arm (Large bore mechanical thrombectomy system vs. catheter-directed therapy)	Hemodynamically stable PE	Currently active trial	Currently active trial
PEERLESS II trial [27]	Double arm (Large bore mechanical thrombectomy system vs. anticoagulation)	Intermediate risk	Currently active trial	Currently active trial

The FLASH registry: The FlowTriever all-comer registry for patient safety and hemodynamics (FLASH) is a multi-center prospective registry evaluating the efficacy and safety of percutaneous mechanical thrombectomy in PE. The FlowTriever System is an U.S. Food and Drug Administration (FDA)-approved device for the treatment of PE that combines aspiration and mechanical thrombus extraction that does not require thrombolytic use and hence reduces the risk of bleeding complications. This registry includes patients with both intermediate- and high-risk PE, which was not included in the FLARE trial. Eight hundred patients were enrolled in this study, with 92.1% diagnosed with intermediate-risk (sub-massive) PE and the remaining 7.9% being diagnosed with high-risk (massive) PE. Around 65% of patients had concomitant deep vein thrombosis, and the sPESI score was 1.6±1.1 [28,23]. 

The MAE at 48 hours in this study was 1.8% (n=14). This included 11 major bleeds (1.4%) and three intraprocedural MAE (0.4%). None of the MAEs had a confirmed relationship with the study device. This low MAE rate is worth noting given the patient population has significant comorbidities, with 65% of patients diagnosed with concomitant DVT. This is similar to the FLARE study, where the MAE rate at 48 hours was 3.8% [21,23]. 

The all-cause mortality rate was 0.3% and 0.8% at 48-hour and 30-day follow-up, respectively, which were not related to the study device. These results show improvement in mortality rates as compared to the PERT registry, which shows up to 30% and 15% mortality (30 days) in high-risk PE and intermediate-risk PE patients [23,29]. 

Results from this registry show that there was a significant improvement in the mean pulmonary artery pressure pre- and post-thrombectomy (from 32.6±9.0 mmHg to 24.9±8.9 mmHg). In addition, patients with pulmonary hypertension at baseline (n=99, 12.7%) showed a significant reduction in systolic pulmonary artery pressure from 78±12.1 to 60.9±15.2 mmHg. There was one death in this patient subset through 30-day follow-up and three MAE (3.1%) within 48 hours of the procedure [29]. 

There was a significant improvement in RV size and function post-procedure. The RV/LV ratio decreased from 1.23± 0.36 to 0.98±0.31 and the RV systolic pressure decreased from 48.9±14.9 to 38.8±14.8 mmHg at 48 hours post-procedure. The proportion of patients with severe RV dysfunction decreased from 29% to 4.7%, and the proportion of patients with no or mild dysfunction increased to 74.5% from 33.9% post-procedure [29]. 

Interim results from the FLASH registry showed a significant on-table reduction in mean PA pressure of 7.6 mmHg (22%, p<0.001) from 34.6± 7 mmHg to 27±8 mmHg. There was a significant decrease in the total pulmonary vascular resistance from 6.1±2.5 to 4.6±2 Woods unit (p<0.001) [26]. These improvements in hemodynamic and RV function were similar to the SEATTLE-II study; however, the key difference noted was the rapid on-table improvement without the risk of bleeding [15,23]. 

The post-procedural hospital stay in this cohort was as low as three days (IQR 2-5 days) as compared to a mean of 8.8 days in the SEATTLE II trial. Similarly, the median post-procedural ICU stay was one night (IQR 1-2 days) in this cohort, and only 37.4% of patients had to be admitted to the ICU [15,23]. 

The FLASH is the largest prospective study in interventional PE, with 800 patients enrolled across 50 centers in the US. These results show that mechanical thrombectomy can be an economically sustainable practice in the long term with lower hospital and ICU stays, lower readmission rates, and a low risk of adverse events. However, since this was a single-arm study, there were no direct comparisons made to other treatment approaches [28,23]. 

## Conclusions

Clinical trials have studied CDT for more than a decade now and have shown that CDT improves the post-procedural RV/LV ratio and has a reduced rate of bleeding episodes and all-cause mortality. Trials like the FLARE have also shown that CDT is more effective in patients with pulmonary disease and results in shorter ICU stays. However, there is a lack of large prospective RCTs studying the effects of CDT in intermediate-high-risk PE patients to determine which patients from this sub-group require CDT both in terms of improving short-term mortality risk and long-term morbidity (such as CTEPH and post-PE syndrome). 

Future clinical trials need to focus on identifying which patient groups will benefit from CDT over anticoagulation and if there are any advantages of using EKOS (US-assisted CDT) over standard CDT. In addition, the scientific community needs to study the health care costs of CDT over traditional treatment, which is relevant for public health systems such as the National Health Service (NHS). CDT may help avoid the most dreaded complication of anticoagulation (ICH and other major bleeding); however, this has not been studied on a large scale previously. 

Lastly, we need standardized guidelines for the use of thrombectomy systems since pulmonary embolism is a complex disease requiring a multifaceted and nuanced treatment approach. In addition, future clinical trials should be designed to answer whether there are any efficacy and safety-related advantages of using the FlowTriever or the Penumbra system (manual vs. motorized pump for aspiration) in PE patients. 
